# Clinical Lectures upon Mental and Nervous Diseases

**Published:** 1869-11

**Authors:** 

**Affiliations:** Sainte Anne, Paris


A MONTHLY RECORD OF

*Medicine, Surgery, and the Collateral Sciences.*

EDITED BY J. ADAMS ALLEN, M.D., LL.D.; AND WALTER HAY, M.D.

VOL. XXVI. — NOVEMBER, 1869. — No. 20.

### Original Translations.

ARTICLE I. — *Clinical Lectures upon Mental and Nervous Diseases; Alcoholism, Alcohol and Absinthe, Absinthic Epilepsy.* By M. MAGNAN, Sainte Anne, Paris.

BEFORE beginning the purely clinical investigation of alcoholic intoxication, either acute or chronic, it is necessary to take into consideration the mode of action of the poison upon the economy. For a long time it has been thought, and it is even now generally believed, that spirituous liquors: wines, brandies, liqueurs, cider, beers, act upon the organism in consequence of the alcohol which they contain, and in direct proportion to the quantity of this substance. Disregarding foreign matters, such as the salts of lead, copper, strychnia, even, introduced fraudulently into these beverages, it is necessary to know the action which pertains to each of the substances entering into the regular composition of drinks.

I shall now proceed to give you the exact details concerning one of the most commonly used spirituous liquors. I refer to Absinthe. Different processes are employed in the fabrication of these products. After having macerated, during a greater or less length of time, different substances in alcohol, the mass may be distilled, and the essential portion of the liquid thus obtained; but, at present, the greater number of the manufacturers prepare the liquor cold, without distillation, they satisfy themselves with the use of certain essences which they mix, in greater or less quantity, with the alcohol. I have procured the formulæ of different manufacturers, and have been able to satisfy myself of the nearly uniform use of the same substance by nearly all of them. The differences concern more especially the quantities and qualities of the alcoholic excipient. The substances which, in addition to alcohol and absinth, enter intothe composition of the absinth liquor are star anise, anise, angelica, *calamus aromaticus*, origanum, sometimes balm, fennel and mint. These three last are added only by some manufacturers.

Now, gentlemen, let us examine what becomes of alcohol in the organism, and what are its effects. Alcohol, according to the theory of Liebig, considered as a respiratory, undergoes in the economy metamorphoses of successive oxydations which terminate, finally, in the production of water and carbonic acid. MM. Bouchardat and Sandras,* by endorsing this doctrine, have rendered it classic; likewise, in 1860, when the remarkable work of MM. Lallemand, Perrin and Duroy† appeared, it was asserted, generally, that alcohol was an alimentary body, and was assimilated.

These authors, you know, combat, directly, the classic opinion, rob alcohol of its elementary properties, but they assign to it a direct and primary action upon the organism, and more particularly upon the nervous centres. They admit that alcohol penetrates into the economy, remains there in its natural state, and is eliminated without undergoing the least modification: that it escapes, as it enters, in the state of alcohol.

In the face of two doctrines so opposite, both based upon investigations carefully conducted, it becomes necessary to examine upon which side the truth is to be found, or even to determine the part to be assigned to each one of these opinions; the more so, as accurate ideas about alcohol will be of great service

* *Annales de Chimie et de Physique.* 1847.

† Lallemand, Perrin et Duroy. 1860. *Du rôle de l'alcool et des anesthetiques dans l'organisme.*

in the establishment of therapeutical indications. Permit me, then, to delay one moment for their discussion.

It may be said that, in favor of the old theory, there is no direct demonstration. This phase of the subject, corresponding to what is admitted about the chemical phenomena of digestion, it seems to be so likewise of them. Efforts have been made to detect in the economy the products of the different transformations assumed by alcohol under the influence of successive oxidations; but the results have not been very conclusive. MM. Bouchardat and Sandras, with great difficulty, succeeded in detecting the existence of one of the intermediate products, acetic acid, in the blood of alcoholized animals; they recognized it by its odor, and by a very slight acid reaction.

On the other hand, Duckel, of Prague, asserted that aldehyde could be detected in the blood; that aldehyde was rapidly transformed, and would give origin to the reproduction of acetates and oxydates; that thus, by a series of duplications, the formation of water and carbonic acid was reached. But these isolated investigations have not, to my knowledge, received the support of recent analysis. Moreover, as the quantities of alcohol eliminated, invariably differ widely from the doses administered, the partizans of the theory of Liebeg are strengthened by the well-known fact; we shall see what is to be concluded from it.

Let us examine, first, the evidence advanced by MM. Lallemand, Perrin and Duroy, in support of their theory. These authors demonstrated, on the one hand, the presence of alcohol, unchanged, in the economy; on the other, its elimination by the different excretory channels, the kidneys, the lungs, the skin.

MM. Bouchardat and Sandras, who had likewise determined the presence of alcohol in the products of the pulmonary exhalation, have detected none of it in the urine; but the majority of experimenters have observed it there. M. Baudot,* in particular, one of the most zealous defenders of the old theory, declares, in one of his analyses, that a man having swallowed three hundred and five cubic centimetres of alcohol, evacuated ten cubic centimetres of it in two kilogrammes of urine passed in about twenty hours. He rests, indeed, upon this scanty elimination by the urine, in

* *Union Médicale*, 1863, 1864. De l'alcool, de sa destruction dans l'organisme.

order to arrive at the conclusion that a large portion of the alcohol must necessarily be destroyed. It has been said, weigh first, weigh afterwards, and then decide. This argument of the scales, gentlemen, is more specious than solid. Doubtless, it is possible to determine, with sufficient accuracy, the quantity of alcohol eliminated by the urine, but the capacity, and especially the duration, of the elimination by the pulmonary and cutaneous channels is entirely ignored.

You see before you a dog plunged, at this moment, into a state of alcoholic intoxication; approach his nostrils, you will receive, at each expiration, puffs of alcoholic vapor.

This elimination will continue with the same degree of intensity during three or four hours; after which it diminishes; but frequently, it is possible, after the expiration of ten or twelve hours from the beginning of the experiment, to determine, by its odor, the presence of alcohol in the expired air; and this, certainly, is not the latest period of the elimination, since alcohol can be detected, we have ascertained it personally, in the organs and in the blood of persons dead three or four days after their last debauch.

At first more active, the elimination diminishes gradually, and terminates by becoming nearly insensible, but it does not the less continue, although its intensity can scarcely be appreciated.

To these direct proofs of the persistence of alcohol in the economy, we will add indirect proofs quite as important. If the alcohol were transformed, the successive oxydations could not be accomplished without inducing modifications of calorification and in the formation of certain products; in other words, we ought to observe an elevation of temperature, and augmentation of carbonic acid, an augmentation likewise of urea; chemical observation and experiment prove directly the contrary.

MM. Lallemand, Perrin and Duroy indicate a diminution of temperature of scarcely a degree, in animals submitted to the action of alcoholic liquors. Edward Smith also mentions a cooling of the body, under the same circumstances. But already — in 1848 — MM. Dumeril and Dumarquay had pointed out a considerable reduction of the temperature of animals submitted to the action of alcohol; and, in one of their experiments, they had succeeded in detecting, at the end of three hours, a difference of 9.°6, in a dog to which they had administered 125 grammes (about 4 ounces) of alcohol.

This cooling is more marked than that resulting from ether or chloroform.

We have personally noted, in our experiments, a depression of 3.°5 in animals plunged into alcoholic intoxication of medium intensity, and insufficient to produce death.

Clinical observation, likewise, supplies us with examples, no less remarkable, of diminution of the temperature of the body under the influence of alcoholic drinks. I owe to the courtesy of M. Duquet, chief of clinic to the faculty, the following report of a case of alcoholic intoxication, in which the depression was carried to the utmost limits. The subject was a female, aged thirty-years, of a vigorous constitution, who, on the third of March, 1869, at 10 A.M., was brought to la Pitié, St. Charles ward, No. 14, under the charge of M. Peter. This woman, after having, on the previous evening, indulged in copious libations (potations?), was lost in the country, in the neighborhood of Ivry. She had passed the night under a freezing, drenching rain; at about three o'clock in the morning, had been seen wandering, by some farm laborers. At six o'clock, she had been found, lying cold and senseless, in a ditch.

On arriving at the hospital, there was complete coma, and considerable superficial coldness; almost complete abolition of sensibility; the arms and legs were agitated by slight convulsive movements, feeble in character, as it were twistings, or rather a sort of crawling in all directions; the force expressed in these movements was considerable; the pulse was full and règular, but infrequent. *The temperature, tested carefully at the moment of inspection, indicated twenty-six degrees* (Centigrade, 55.12° Fahrenheit), *in the axilla and vagina.*

The patient placed in bed, was warmed by means of hot bricks; and stimulating drinks were administered. Gradually, the convulsive phenomena ceased, the temperature of the body was insensibly elevated, and the patient recovered consciousness at half-past four P.M.

From this moment, all the functions were reëstablished rapidly, and her return to health was affected sufficiently to admit of her discharge from the hospital on the following day.

M. Hirne, clinical statistician, registered the temperature at different intervals, from the first examination, which gave 26°. The scale below exhibits the gradual elevation:

| *Hour.* | *Vaginal Temperature.* | *Axillary Temperature.* |
|---|---|---|
| 11.30 | 27.°9 | 27.°9 |
| 12.30 | 28.°7 | 28.°6 |
| 12.45 | 30.°4 | 30.° |
| 1.15 | 30.°9 | 31.°1 |
| 3.15 | 34.°4 | 34.°3 |
| 4.20 | | 36.°3 |

While assigning even a very important part to the action of cold, it is impossible to avoid recognizing the influence of spirituous liquors in this diminution of nearly 11 degrees.

You perceive the demonstration is complete, as far as regards temperature; on the other hand, the investigations of Bocker* of Vierordt, of Lehman, of MM. Perrin show that the quantity of carbonic acid is less, and that the diminution is in direct ratio to the dose of alcohol imbibed. Finally, the urine does not contain an increased quantity of urea. Certain observers, Bokes, especially, notice even a slight diminution.

You perceive, gentlemen, abundant evidence in favor of the new theory, and it would be a strange error to consider alcohol eliminated as a complete superfluity, as a simple excess of which the economy should be freed. We do not believe, however, that it is necessary to repeat, altogether, the idea of the possible transformation of a portion of the alcohol. The disagreeable acescence which accompanies drunkenness, should be alone sufficient to indicate changes occurring, even before its absorption in the circulation. To sum up, the principal fact, that which occupies the most prominent position, is the passage of alcohol through the organism, and its escape in a natural state through the different excretory channels; hence its invariably uniform action, direct and immediate, upon the different organs, and more especially upon the nervous centres.

A secondary, accessory fact is the transformation of a small portion only of the digested alcohol.

Knowing, now, the manner in which alcohol acts, let us

*Archives General de Médècine. 1849.

investigate the immediate phenomena to which it gives rise, in doses slightly increased.

Under its influence, muscular action, at the end of some minutes, escapes from the control of the will; the animal staggers; the paws overlap, especially the hinder ones, one or the other gives way, and the body falls upon its side; at first, the animal is able to raise himself, and resumes his equilibrium, which he soon loses again in the same manner; next, his two hind paws bend, and are drawn up under the animal, who, raised upon his fore-paws, endeavors to crawl, dragging along with difficulty his posterior extremities.

Gradually, the paralysis overtakes the posterior portions of the body; the animal presents a condition of complete relaxation, and is soon plunged into a comatose sleep; if awakened, he relapses like an inert mass, the limbs pendulous, and the head likewise, all elasticity being abolished; next alvine evacuations and vomitings occur, and the subject, completely intoxicated, remains prostrate in the midst of his evacuations.

The thermometer placed in the rectum indicates a considerable reduction of temperature; the breath exhales a decided alcoholic odor.

The urine of animals, submitted temporarily to the action of alcohol, presents no traces of albumen. These phenomena persist during variable periods, which may be extended to three or four hours, and even more; after which the return to health proceeds slowly, when the animal, on awakening, resumes, as soon as he can, his habitual movements.

Under some circumstances, all is not over; pulmonary or cerebral complications ensue, which may become rapidly fatal.

It has been sought to determine if the different varieties of alcohol had different effects. Analogous phenomena have been determined for all, but varying in intensity according to the quality of the alcohol. Thus M. Cros, in his thesis, shows, by experiments upon rabbits, pigeons, and upon men, that amylic alcohol, in equal doses, develops much more decided phenomena than vinous alcohol; but the general character of the symptoms remains nearly the same. Its topical effect upon the digestive canal has been observed to be irritative and more energetic.

A very important fact, gentlemen, to which I desire to direct your attention in an especial manner, is, that in a considerable number of experiments made with alcohol, there has been no recorded observation of the appearance of epileptic or epileptiform convulsions. You will hence perceive the necessity of seeking elsewhere than in the immediate effect of alcohol, the cause of epilepsy in acute alcoholism.

M. Magnan presented a series of different animals submitted to alcoholic intoxication in various degrees. These animals evinced the phenomena which have just been discussed. In addition, there was remarked, in the case of a guinea-pig, besides this paraplegia, slight tremor of the paws; this tremor which was not perceptible in every case, ceased generally when the animal was in a comatose sleep.

We now perceive, continued M. Magnan, the anatomical modifications to which acute alcoholic intoxication gives origin.

For a long time, hitherto, the presence of alcohol has been detected in the nervous centres. Ogston discovered it in the brain of a drunken woman; M. Jardien and others have observed the alcoholic odor exhaled by the brain of persons who have died in a drunken condition. But the investigations of M. Lallemand, Perrin and Duroy have contributed especially to establish a clear demonstration of the presence of alcohol in the different organs, and in the blood. For our own part, in 1864, we had, at the Bicêtre, the opportunity to repeat these analyses, with the coöperation of M. Belin, pharmaceutical interne. He determined in man, and also in animals submitted to experiment, the presence of alcohol in the brain and spinal cord, in the liver, in the blood, and likewise, but in very small quantity, in the lungs and in the kidneys.

From the analyses made at St. Anne, with M. Chatenier, pharmaceutical interne, we have arrived at the same result. We have obtained from different organs, by distillation, products having the physical and chemical properties of alcohol. With the test-liquor,

| | |
|---|---|
| Bichromate of Potass - - - - - - - | 1 |
| Sulphuric acid - - - - - - - - - - | 30 |

A magnificent emerald green color is obtained, chromic acid gives a black tint; ammoniac a blackish-brown deposit. The distilled liquor exhaled an alcoholic odor, and the vapors took fire from the light of a candle, producing bluish flames, like ordinary alcohol.

Spirituous liquors are not distributed equally and uniformly throughout the organism; certain organs appear to have a particular affinity for these substances, and store them up in greater quantity. It is well to observe, however, that the channel of absorption has a definite influence upon the more particular accumulation of alcohol in certain organs. Thus, when absorption was effected through the pulmonary mucous surface, or through the veins, the poison is found in greater quantity in the nervous centres. When, on the contrary, the liquids are introduced into the stomach, the portal vein carries toward the liver the substances contained in the gastric cavity, and the liver then becomes richest in alcohol. The blood falls into the third rank; then the kidneys, and the lungs.

The cerebro-spinal membranes are injected, frequently suggillations are perceived, little sanguineous infiltrations into the thickness of the pia-meter; sometimes even a little blood spread out in a layer upon the surface of the arachnoid.

These meningial hæmorrhages have appeared to me the more common in men than in animals. The brain and the spinal cord are generally injected; the gray substance, whether at the periphery or the centre, assumes a rosy color, more or less deep, and, under some circumstances, a very remarkable tint, like the meat of ham.

The mucous membrane of the stomach presents, almost always, in animals, a vivid injection, sometimes little ecchymotic patches, and sometimes hæmorrhages, either under the membrane, within its thickness, or upon the surface; and then small ulcerations may be discovered, covered with blackish clots.

The intestines present a variable degree of injection, which is arrested, generally, in the upper portion of the small intestine. It is not rare to find in the lungs reddish patches, congested, sometimes also with apopleptic points; but these lesions are always disseminated.

The liver and the kidneys are injected, but they are very rarely the seat of hæmorrhages.

§ II.—When an animal is submitted to the prolonged action of alcohol, the phenomena which we have just indicated will be seen to supervene after each administration of the poison, and, in addition, certain symptoms, which are not met with in simple drunkenness.

A vigorous dog, two and a half years old, weighing 16 killogrammes, took, during about two months, a daily dose of 60 grammes of alcohol. Each time, eight to fifteen minutes after the injection of the toxic liquid, the animal staggered, falls collapsed, in a state of complete resolution, with relaxation of the sphincters; he fell into a comatose sleep, of which the duration from the commencement scarcely exceeded two or three hours. At the beginning of the poisoning process, immediately on awakening, the normal state returned quite rapidly; but from the second month, in proportion as the chronic intoxication progressed, the animal preserved always a slight hebetude.

A symptom especially worthy of arresting the attention, and which suggests a certain resemblance to *delirium tremens* in man, is the appearance of tremor, which, manifesting itself first in the paws, gradually becomes general, involving the muscles of the neck and of the trunk, in the last fortnight. This tremor, which was persistent during a greater portion of the day, did not entirely disappear during the comatose sleep. Every morning the stomach was found to be filled with mucus; the cleansing of the orifice of the gastric canula, permitted the escape therefrom of a stream of liquid, viscous and adhesive, analogous to the pituitary secretion of drunkards. Upon several occasions, during the course of these experiments, I increased the dose of alcohol to 70 and even to 75 grammes. This addition of from ten to fifteen grammes to the habitual dose sufficed to induce symptoms whose intensity was altogether disproportioned to this slight additional quantity.

A degree of saturation had been reached which could not be exceeded. In man, as is well known, the same condition exists.

Autopsy shows a thickening of the gastric tunics. The mucous surface, of a reddish-brown, is covered with a layer of thick tenacious, vitreous mucus, streaked with blood at intervals; upon cleansing this surface, by means of a stream of water, small ulcerations were seen below, with irregular borders, and in some places, cicatrized portions in the form of irregular, grayish patches. In the thickness of the mucous membrane, sanguineous infiltrations were observed, some spread out in sheets, others collected in little foci. The liver presented a general yellowish tint, studded with more deeply colored points in the centre of the lobules. The kidneys had already assumed incipient fatty degeneration, especially in the cortical layer, and in the columns of Bertini. The cavities of the heart, especially those of the right side, and the venæ cavæ were distended, and filled with blackish clots.

The lungs exhibited some sub-pleural ecchymoses. The membranes, slightly œdematous, presented a rosy color at the level of the chiasma. The cerebral substance was a little injected. Chemical analysis revealed the presence of alcohol in the organs, and the microscopic examination permitted a more thorough investigation of the fatty degeneration of the liver and of the kidneys. Although this dog had not lived a sufficiently long time to manifest the very advanced lesions of chronic alcoholism, nevertheless, what we discovered sufficed to give us an idea of those lesions which presented themselves in the animal, with characters analogous to those habitually encountered in man.

In an oral communication, M. Leidersdorf, of Vienna, informed me that Dr. Kremiansky, having fed young dogs with alcoholic soups, had succeeded, in the course of a few months, in producing in these animals cerebral pachymeningitis, a lesion which is well known to be not infrequent in chronic alcoholism.

